# Inheritance of Mesotrione Resistance in an *Amaranthus tuberculatus* (var. *rudis*) Population from Nebraska, USA

**DOI:** 10.3389/fpls.2018.00060

**Published:** 2018-02-02

**Authors:** Maxwel C. Oliveira, Todd A. Gaines, Amit J. Jhala, Stevan Z. Knezevic

**Affiliations:** ^1^Department of Agronomy and Horticulture, University of Nebraska-Lincoln, Concord, NE, United States; ^2^Department of Bioagricultural Sciences and Pest Management, Colorado State University, Fort Collins, CO, United States; ^3^Department of Agronomy and Horticulture, University of Nebraska-Lincoln, Lincoln, NE, United States

**Keywords:** waterhemp, herbicide resistance evolution, polygenic, 4-hydroxyphenylpyruvate dioxygenase, metabolism-based resistance

## Abstract

A population of *Amaranthus tuberculatus* (var. *rudis*) evolved resistance to 4-hydroxyphenylpyruvate dioxygenase (HPPD) inhibitor herbicides (mesotrione, tembotrione, and topramezone) in Nebraska. The level of resistance was the highest to mesotrione, and the mechanism of resistance in this population is metabolism-based likely via cytochrome P450 enzymes. The increasing number of weeds resistant to herbicides warrants studies on the ecology and evolutionary factors contributing for resistance evolution, including inheritance of resistance traits. In this study, we investigated the genetic control of mesotrione resistance in an *A. tuberculatus* population from Nebraska, USA. Results showed that reciprocal crosses in the F1 families exhibited nuclear inheritance, which allows pollen movement carrying herbicide resistance alleles. The mode of inheritance varied from incomplete recessive to incomplete dominance depending upon the F1 family. Observed segregation patterns for the majority of the F2 and back-cross susceptible (BC/S) families did not fit to a single major gene model. Therefore, multiple genes are likely to confer metabolism-based mesotrione resistance in this *A. tuberculatus* population from Nebraska. The results of this study aid to understand the genetics and inheritance of a non-target-site based mesotrione resistant *A. tuberculatus* population from Nebraska, USA.

## Introduction

Waterhemp [*Amaranthus tuberculatus* (var. *rudis*)] is a classic example of herbicide resistance evolution. *A. tuberculatus* is a native species of the Midwestern United States, and it has become a predominant weed in corn-soybean cropping systems (Owen, 2008; Waselkov and Olsen, 2014). The biology of *A. tuberculatus* is an important factor contributing to its adaptation in row-crop production systems. The dioecious nature of *A. tuberculatus* enforces cross-pollination and the potential for gene flow of herbicide resistance genes (Trucco et al., 2005, 2006; Sarangi et al., 2017). Moreover, a single *A. tuberculatus* female plant can produce over a million seeds depending on density (Hartzler et al., 2004); therefore, herbicide resistance may evolve and spread faster in *A. tuberculatus* than other monoecious weedy *Amaranthus*. *A. tuberculatus* is ranked among the worst herbicide resistant weeds (Tranel et al., 2011; Heap, 2014). As of 2017, populations of *A. tuberculatus* have evolved resistance to six herbicide sites-of-action (SOA) in the United States (Heap, 2017).

Herbicides inhibiting 4-hydroxyphenylpyruvate dioxygenase (HPPD) represent the latest introduced SOA for weed control in corn, commercialized in the late 1980's (Mitchell et al., 2001; Duke, 2012). Mesotrione, an HPPD-inhibitor herbicide, was introduced as an effective preemergence (PRE) and postemergence (POST) herbicide for controlling various broadleaf weeds, including *A. tuberculatus* (Mitchell et al., 2001; Sutton et al., 2002). However, resistance to mesotrione evolved recently (Hausman et al., 2011; McMullan and Green, 2011), and it is increasing across the north-central United States (Schultz et al., 2015). The persistence and adaptation of HPPD-inhibitor herbicide-resistant *A. tuberculatus* populations to cropping-systems is a concern. There will be a potential increased use of these herbicides by the use of transgenic HPPD-inhibitor-resistant crops in the United States. Therefore, tactics are needed to minimize the evolution of resistance to this herbicide group.

The mechanism of herbicide resistance discovered in *A. tuberculatus* varies according to the herbicide SOA, which can be either target site resistance (TSR) or non-target site resistance (NTSR). Target-site amino acid substitutions, codon deletion, and gene amplification are the major mechanisms of TSR (Patzoldt and Tranel, 2007; Thinglum et al., 2011; Lorentz et al., 2014; Chatham et al., 2015), which are often caused by a dominant gene in a single locus (Délye et al., 2013). In contrast, NTSR includes mechanisms that are not TSR, frequently resulting from multiple genes conferring reduced herbicide penetration, herbicide differential translocation, and enhanced herbicide metabolism (Powles and Yu, 2010; Délye, 2013; Délye et al., 2013). Enhanced metabolism (NTSR) is the only reported mechanism of mesotrione resistance in *A. tuberculatus* (Ma et al., 2013; Kaundun et al., 2017).

A population of *A. tuberculatus* (hereafter referred as R) has evolved resistance to POST-applied HPPD-inhibitor herbicides (mesotrione, tembotrione, and topramezone) in a corn/soybean production system in northeast Nebraska (Oliveira et al., 2017a). Mesotrione detoxification to 4-hydroxymesotrione has been confirmed as the mechanism of resistance in this population (Kaundun et al., 2017). Further research characterized the role of cytochrome P450 enzymes in this R population, as malathion (a cytochrome P450 inhibitor) did not synergize mesotrione (Oliveira et al., 2017b). This result is different from what was previously reported in a different mesotrione resistant *A. tuberculatus* population from Illinois, in which malathion synergized mesotrione (Ma et al., 2013). It is likely that different P450 enzymes are endowing mesotrione resistance in different *A. tuberculatus* populations. Therefore, empirical studies are needed to understand the eco-evolutionary dynamics causing weed evolution (Neve et al., 2009, 2014). For example, inheritance studies can improve our knowledge of the genetic structure of weed populations under herbicide selection and aid to create appropriate herbicide resistance simulation models (Neve et al., 2014; Renton et al., 2014; Menalled et al., 2016).

Inheritance of herbicide alleles contributing to pesticide (e.g., herbicide) resistance can vary with different genetic backgrounds and dose environment (Ffrench-Constant et al., 2004; Neve et al., 2014). Thus, the objective of this study was to determine the mode of inheritance and number of alleles controlling mesotrione resistance in the R population from Nebraska with two experiments: (1) dose-response studies with parental [mesotrione R and susceptible (S)] and F1 families generated from the S × R cross; and (2) segregation studies in pseudo-F2 and back cross (BC) families with low, recommended, and high mesotrione dose.

## Methods

### Plant material and growth condition

Two *A. tuberculatus* were the originating populations in this study. This species is an obligate outcrosser so inbred lines could not be developed to establish parents for the inheritance studies. The seeds of the R population were collected in 2014 from a field in Platte County, NE, where mesotrione resistance was confirmed (Oliveira et al., 2017a). The S population seeds were collected from Dixon County, NE; this population was known to have high sensitivity to mesotrione. For both populations, seeds were collected from at least 20 plants. The seeds were cleaned and stored separately (R and S populations) at 5 C until used in the greenhouse study in 2015 and 2016 at the University of Nebraska-Lincoln. Seeds were planted in 1200 cm^3^ plastic pots for pairwise crosses containing peat:soil:sand:vermiculite (4:2:2:2) potting mix. In addition, seeds were planted in 164 cm^3^ cone-tainers (Ray Leach “Cone-tainer” SC10®, Stuewe and Sons Inc, Tangent, OR, USA) for dose-response and segregation analysis with the same potting mix described. Plants were supplied with adequate water and kept under greenhouse conditions at 28/20 C day/night temperature with 80% relative humidity. In addition, twice per week, plants were fertilized with 3 mg of NPK (20-10-20 Peters® Professional, JR Peters Inc., Allentown, PA, USA) for each 100 cm^3^ of the potting mix until plants were 8–10 cm tall. Artificial lighting was provided using metal halide lamps (600-μmol photons m^−2^ s^−1^) to ensure a 15-h photoperiod.

### Generations of F1, pseudo-F2, and back-cross families

**Figure 1:**
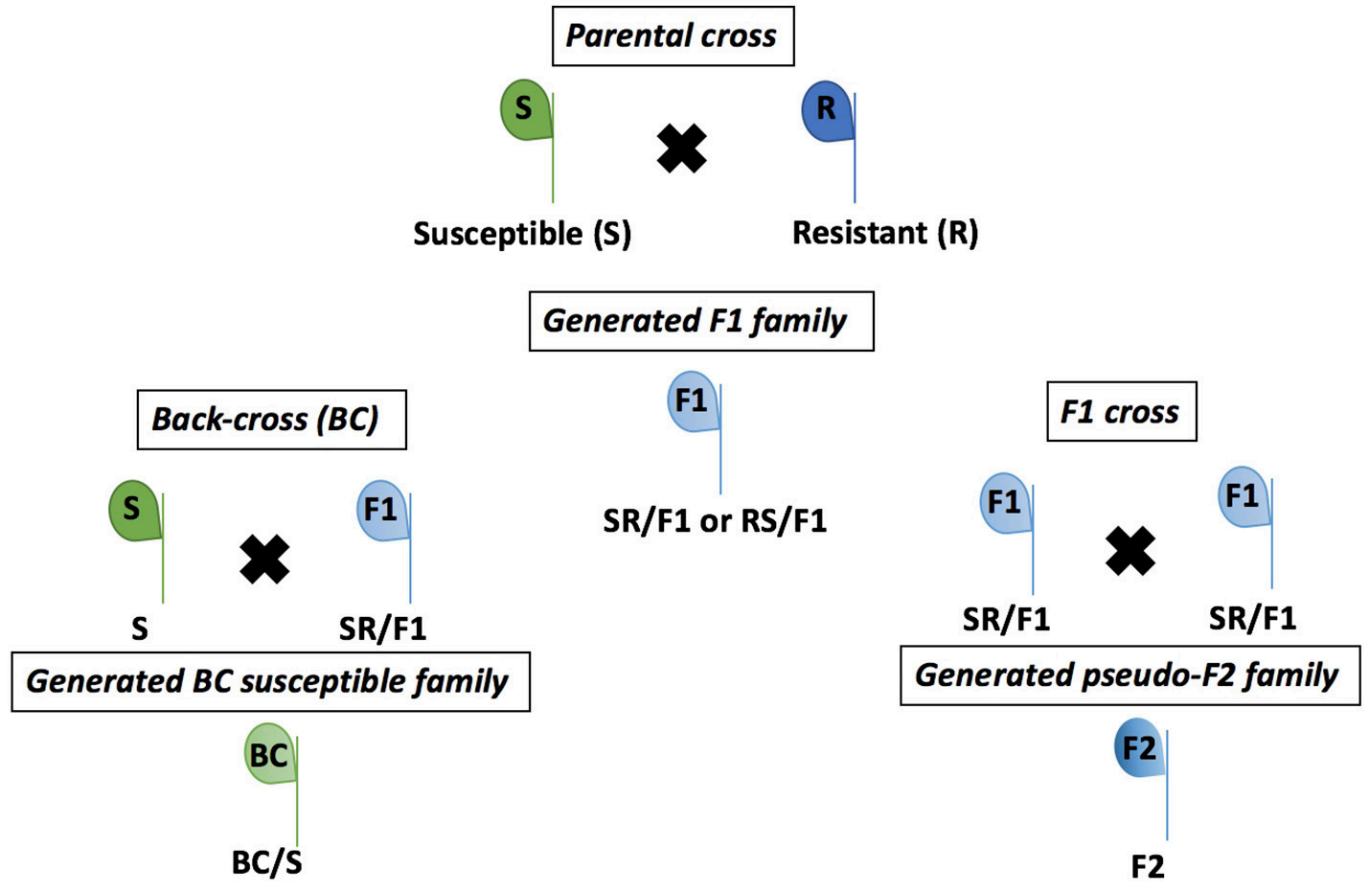
Crosses of *Amaranthus tuberculatus* populations (R and S) to generate F1, F2, and BC/S families conducted in greenhouse at the University of Nebraska-Lincoln.

The R population was grown from seeds in a greenhouse and selected with a POST application of the recommended rate of 105 g ai ha^−1^ of mesotrione (Callisto®, Syngenta Crop Protection, Research Triangle Park, NC, USA) when R seedlings were 10–15 cm tall. The recommended mesotrione rate caused low injury (<30%) on R plants. Therefore, the R plants were kept for individual pairwise crosses with the S plants. Single S and R plants growing in pots were paired according to floral synchrony and enclosed with pollination bags (100 by 185-cm) to exclude external pollen. Male plant inflorescences were gently shaken in the morning during pollination period (~3 weeks) to ensure cross-fertilization between S and R individuals. At maturity, seeds were collected from each female plant, cleaned, and bagged separately, then designated as F1 families (Figure 1).

The F1 families were derived from 20 parental crosses. Four F1 families were advanced to the next generation of crosses (Table S1), including three S × R and one R × S (female × male). The F1 plants were termed RS/F1-5, SR/F1-8, SR/F1-9, and SR/F1-13 (Table S1). The R parent of the SR/F1-13 family was derived from an R × R cross of the R population seeds, and the R × R cross was performed under greenhouse conditions, similarly as described for the S × R cross. The remaining F1 families were derived from field collected R and S seeds. The F1 plants were treated with 105 g ai ha^−1^ of mesotrione when plants were 10 to 15 cm tall. The F1 plants survived with variable mesotrione injury (data not shown) confirming that they were crosses between R and S. *A. tuberculatus* is dioecious, preventing F1 self-pollination to produce true F2 plants (Figure 1). Therefore, the F1 family SR/F1-9 and SR/F1-13 individuals were separately cross-pollinated using the procedures described above to make pseudo-F2 plants (hereinafter referred as F2). The F2 plants were designed F2-9 (from SR/F1-9) and F2-13 (from SR/F1-13). In addition, F1 male plants were also allowed to pollinate the parental susceptible female plants (S) to produce backcross susceptible (BC/S) families (Figure 1). Three BC/S families were made and designed BC-8/S (from SR/F1-8), BC-9/S (from SR/F1-9), and BC-13/S (from SR/F1-13; Table S1).

### Dose-response of mesotrione in F1 families and parental population

The greenhouse experiments were set in completely randomized design with four replications and repeated twice in 2016 at the University of Nebraska-Lincoln. Mesotrione dose-response studies were conducted separately with R and S (parent); and F1 families, including RS/F1-5, SR/F1-9, and SR/F1-13.

The mesotrione dose was 0, 0.125×, 0.25×, 0.5×, 1×, 2×, 4×, 8×, 16×, where 1× represents 105 g ai ha^−1^ (labeled use rate of mesotrione). Mesotrione was mixed with 1% (v/v) crop oil concentrate (Agri-Dex®, Helena Chemical Co, Collierville, TN, USA) and 20.5 g L^−1^ of ammonium sulfate (DSM Chemicals North America Inc., Augusta, GA, USA). Herbicide treatments were applied with a single tip chamber sprayer (DeVries Manufacturing Corp, Hollandale, MN, USA) fitted with an 8001 E nozzle (Spraying Systems Co., North Avenue, Wheaton, IL 60139), calibrated to deliver 140 L ha^−1^ spray volume at 210 kPa at a speed of 3.7 km h^−1^. Control was assessed visually 21 d after treatment (DAT) using a scale of 0 to 100% (where 0 indicates no injury and 100 indicates plant death). Control ratings were based on symptoms such as bleaching, necrosis, and stunting of plants compared to non-treated control plants. Aboveground biomass was harvested at 21 DAT from each experimental unit and oven-dried at 65 C until reaching constant weight before the weight of biomass (g plant^−1^) was recorded.

The effective mesotrione doses needed to control and reduce biomass by 50% (ED_50_) and 90% (ED_90_) for the parental and F1 families were determined using the three-parameter log-logistic curve of the drc package of the R statistical software (Knezevic et al., 2007).

(1) Y=d/1+exp{b[log(x)−log(e)]}

In this model, Y is the control (%) or biomass reduction (g plant^−1^), *d* is the upper limit, and *e* represents the ED_50_ value relative to *d*. The parameter *b* is the relative slope around the parameter *e*, and *x* is the mesotrione dose in g ai ha^−1^.

The resistance level was calculated by dividing the effective dose for providing 50% control or 50% biomass reduction (ED_50_) of the R or F1 populations by the ED_50_ of the S population. The resistance level indices for the respective ED_50_ between the R or F1, and the S were compared using the *EDcomp* function of package drc in R software (Ritz and Streibig, 2005). The *EDcomp* function compares the ratio of ED_50_ using t-statistics, where *P*-value < 0.05 indicates that herbicide ED_50_ values are different between the R or F1 and the S.

The degree of dominance (*D*) was calculated following the formula

(2) $$D=[(2X_{2}-X_{1}-X_{3})/(X_{1}-X_{3})]$$

Where *D* is the degree of dominance and *X*_1_, *X*_2_, and *X*_3_ are the log(ED_50_) of R, F1, and S population, respectively. When *D* = 0, the F1 has a resistance level that is the mean of the R and S (additive). When *D* = 1 there is an indication of complete dominance, 0 < *D* < 1 supports the model of incomplete dominance, −1 < *D* < 0 of incomplete recessive, and *D* = −1 of complete recessive (Stone, 1968).

### Mesotrione segregation in *Amaranthus tuberculatus* F2 and BC/S families

The R and S parent, SR/F1 (SR/F1-8, SR/F1-9, and SR/F1-13), F2 (F2-9 and F2-13) and BC/S (BC-8/S, BC-9/S, and BC-13/S) families were treated with three mesotrione doses attempting to discriminate S from R plants. These doses were 26, 105, and 420 g ai ha^−1^, which represent 0.25×, 1×, and 4× the recommended rate, respectively. The 0.25× dose was chosen to assess segregation at a below-label dose. The 1× dose was chosen to evaluate segregation at recommended label dose at which plants are selected under field conditions. The higher dose (4×) was selected to likely allow mesotrione resistance segregation to occur with minimal interference of minor resistance loci (Busi et al., 2014). There were two runs (repetitions in time) of this experiment for each mesotrione dose, and each run included BC/S and F2 populations represented by 29 to 98, and 56 to 98 plants, respectively. In addition, parent (R and S) populations were represented by 8 to 17 plants, and F1 populations were represented by 8 to 24 plants. A total of 2,908 *A. tuberculatus* plants were screened with mesotrione in this study. Herbicide application was the same as described above in dose-response studies. At 21 DAT, all populations were visually evaluated and assessed as dead or alive. Alive plants were separated into different injury level groups, including low (<40%), medium (41 to 79%), high (80 to 98%), and dead (>98%; Figure S1). Plant aboveground biomass was harvested, oven-dried until constant weight, and biomass (g plant^−1^) was recorded. The plant biomass and injury level for parental, SR/F1, F2, and BC/S families were represented in violin plots with a rotated kernel density plot using package *ggplot2* in R statistical software (Wickham, 2009).

The experimental null hypothesis was that mesotrione resistance segregates as controlled by a single major gene (one locus). The segregation analysis in F2 and BC/S families was based on the observed survival ratio (alive/total treated plants) compared to expected survival assuming one gene locus segregation (Table S2). For the F2 and BC/S families, the expected number of surviving plants was described by the following equations (Tabashnik, 1991; Busi et al., 2013; Han et al., 2014):

F2: One gene locus model (1R:2F1:1S),

(3) Exp. F2=0.25 × Obs R+0.5 × Obs F1+0.25 × Obs S

BC/S: One gene locus model (1F1:1S),

(4) Exp. BC/S=0.5 × (F1+S)

where R, F1, and S are the number of observed surviving plants of the R, F1, and S families at each mesotrione dose (26, 105, and 420 g ai ha^−1^). Thus, for each dose, the expected number of survivors for F2 and BC/S populations was calculated with the total number of treated plants multiplied by the theoretical one locus segregation ratio for F2 and BC/S families.

A chi-square goodness-of-fit test (χ^2^) was used to compare the observed and expected plant survival based on a one locus segregation model. The *P*-values were obtained to indicate the probability in rejecting the null hypothesis for F2 and BC/S families at one locus segragation. For example, for one locus segregation, the null hypothesis (H_0_) is that the BC segregates as 0.5F1:0.5S. The significant level is α = 0.05 and if *P*-value < 0.05, the null hypothesis is rejected.

## Results

### Mode of inheritance of mesotrione resistance

**Figure 2:**
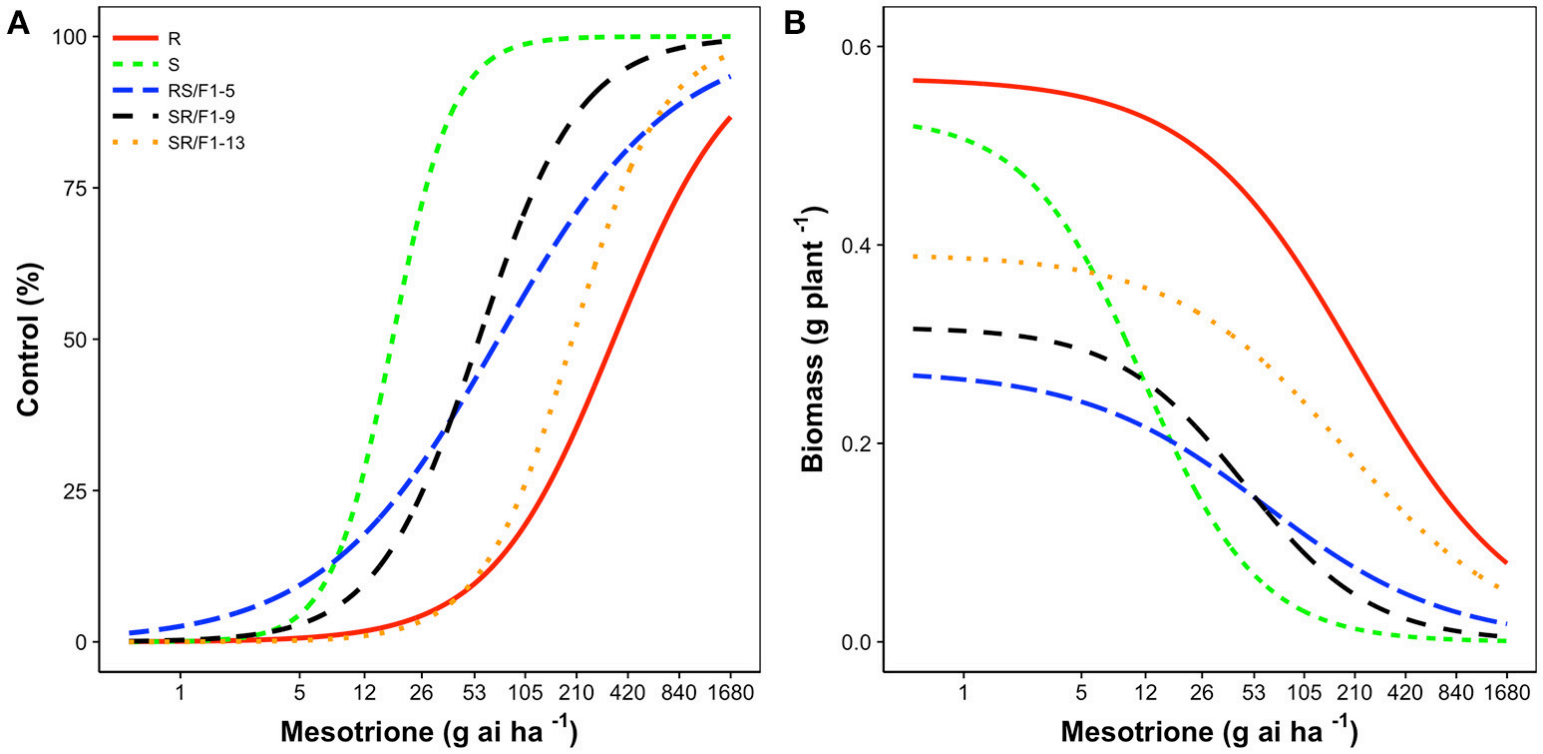
Mesotrione dose-response on **(A)** Control (%) and **(B)** Biomass (g plant^−1^) of *Amaranthus tuberculatus* families (R and S parent populations; and F1 families, RS/F1-5, SR/F1-9, and SR/F1-13) conducted in greenhouse at the University of Nebraska-Lincoln.

The R population displayed a high level of resistance whereas S was susceptible (Figures 2A,B). The level of resistance of R compared to S was 19-fold based on ED_50_ values (Table 1). The generated F1 families provided an intermediate and relatively lower level of resistance (3-4-fold) based on ED_50_ values, except the SR/F1-13 family (11-fold). The biomass (g plant^−1^) of parental and F1 families corroborated with visual control, resulting in similar ED_50_, ED_90_, and resistance levels (Table 2).

**Table 1:** Estimated parameters (*b, d*, and *e*) and effective dose to control 90% (ED_90_) of *Amaranthus tuberculatus* (R and S parent populations; and F1 families, RS/F1-5, SR/F1-9, and SR/F1-13) conducted in greenhouse at the University of Nebraska-Lincoln.

**Population^a^**	**Parameter^b^**			**ED_90_ (±SE)**	***P*-value^c^**	**Resistance level^d^**
	***b* (±SE)**	***d* (±SE)**	***e* (ED_50_) (±SE)**			
			**g ai ha**^−1^			
S	−2.4 (0.2)	100	18 (1)	43 (4)	–	–
R	−1.2 (0.1)	100	348 (19)	2199 (257)	0.00	19
RS/F1-5	−0.8 (0.0)	100	73 (4)	981 (143)	0.00	4
SR/F1-9	−1.5 (0.1)	100	56 (2)	255 (27)	0.00	3
SR/F1-13	−1.6 (0.1)	100	198 (9)	759 (80)	0.00	11

a *S, 4-hydroxyphenylpyruvate dioxygenase (HPPD)-inhibitor herbicide-susceptible A. tuberculatus collected from a field in Dixon County, NE in 2014; HPPD-R, 4-hydroxyphenylpyruvate dioxygenase (HPPD)-inhibiting herbicide-resistant A. tuberculatus collected from a field in Platte County, NE in 2014. RS/F1-5, SR/F1-9, and SR/F1-13 are crosses from SxR parents made under greenhouses conditions*.

b *b, the slope; d, the upper limit (locked at 100); and e (ED_50_), the inflection point relative to the upper limit. The ED_50_ is an effective dose of mesotrione needed to reach 50% HPPD-R control. SE, standard error*.

c *R and F1 families vs. S population t-statistics comparison of e (ED_50_), P-value > 0.05 means non-significant difference between treatments*.

d *Resistance level was calculated dividing e (ED_50_) value of R and F1 families by S population*.

**Table 2:** Estimated parameters (*b, d*, and *e*) and effective dose to reduce biomass (g plant^−1^) 90% (ED_90_) of *Amaranthus tuberculatus* (R and S parent populations; and F1 families, RS/F1-5, SR/F1-9, and SR/F1-13) conducted in greenhouse at the University of Nebraska-Lincoln.

**Population^a^**	**Parameter^b^**			**ED_90_ (±SE)**	***P*-value^c^**	**Resistance level^d^**
	***b* (±SE)**	***d* (±SE)**	***e* (ED_50_) (±SE)**			
	**Biomass (g plant**^−1^**)**		**g ai ha**^−1^			
S	1.3 (0.2)	0.5 (0.0)	12 (1)	66 (12)	–	–
R	0.9 (0.1)	0.6 (0.0)	216 (23)	2665 (408)	0.0	18
RS/F1-5	0.8 (0.1)	0.3 (0.0)	62 (15)	956 (340)	0.0	5
SR/F1-9	1.2 (0.2)	0.3 (0.0)	47 (7)	314 (86)	0.0	4
SR/F1-13	1.0 (0.1)	0.4 (0.0)	183 (31)	2354 (686)	0.0	15

a *S, 4-hydroxyphenylpyruvate dioxygenase (HPPD)-inhibitor herbicide-susceptible A. tuberculatus collected from a field in Dixon County, NE in 2014; HPPD-R, 4-hydroxyphenylpyruvate dioxygenase (HPPD)-inhibiting herbicide-resistant A. tuberculatus collected from a field in Platte County, NE in 2014. RS/F1-5, SR/F1-9, and SR/F1-13 are crosses from SxR parents made under greenhouses conditions*.

b *b, the slope; d, the upper limit (locked at 100); and e (ED_50_), the inflection point relative to the upper limit. The ED_50_ is an effective dose of mesotrione needed to reach 50% HPPD-R control. SE, standard error*.

c *R and F1 families vs. S population t-statistics comparison of e (ED_50_), P-value > 0.05 means non-significant difference between treatments*.

d *Resistance level was calculated dividing e (ED_50_) value of R and F1 families by S population*.

The F1 families expressed a variable degree of dominance (D). For example, based on control, the degree of dominance of SR/F1-13 family was 0.62 (Table 3). In contrast, the SR/F1-9 family had a negative degree of dominance (*D* = −0.22). The RS/F1-5 family had a degree of dominance close to zero (*D* = −0.04). The pooled-F1 family resulted in *D* = 0.11. Similar degree of dominance trend was also observed with plant response to mesotrione based on biomass (g plant^−1^) of the F1 families (Table 3).

**Table 3:** Degree of dominance based on logarithm of parameter *e* (ED_50_) control and biomass (g plant^−1^) of *Amaranthus tuberculatus* (R and S parent populations; and F1 families, RS/F1-5, SR/F1-9, and SR/F1-13) conducted in greenhouse at the University of Nebraska-Lincoln.

**Population^a^**	**Control**	**Degree of dominance^b^**	**Biomass**	**Degree of dominance^b^**
	**log(ED**_50_**)**		**log(ED**_50_**)**	
R	5.85	–	5.37	–
S	2.86	–	2.45	–
RS/F1-5	4.29	−0.04	4.18	0.14
SR/F1-9	4.03	−0.22	3.84	−0.05
SR/F1-13	5.29	0.62	5.21	0.88
F1-pooled	4.53	0.11	4.39	0.32

a *S, 4-hydroxyphenylpyruvate dioxygenase (HPPD)-inhibitor herbicide-susceptible A. tuberculatus collected from a field in Dixon County, NE in 2014; HPPD-R, 4-hydroxyphenylpyruvate dioxygenase (HPPD)-inhibiting herbicide-resistant A. tuberculatus collected from a field in Platte County, NE in 2014. RS/F1-5, SR/F1-9, and SR/F1-13 are crosses from SxR parents made under greenhouses conditions*.

b *Degree of dominance was calculated using the formula D = [(2X_2_-X_1_-X_3_)/(X_1_-X_3_)], where X1, X2, and X3 represent the e (ED_50_) values of R, S, and respective F1 family*.

### Mesotrione segregation in *Amaranthus tuberculatus* F2 and BC/S families

The segregation at below-label mesotrione dose (26 g ai ha^−1^) resulted in 77% survival of S plants when averaging both runs, and survived S plants showed high injury (>80%; Figure 2A). In addition, the 26 g ai ha^−1^ resulted in high survival rate (≥87%) of the SR/F1, F2, and BC/S families across runs. Therefore, this dose was not the best to describe a segregation analysis for this R population. Nonetheless, a single major gene model would not fit (*P*-value < 0.05) the majority of F2 and BC/S families (Table 4).

**Table 4:** Phenotypic resistance segregation observed in two pseudo-F2 (F2) and three back-cross susceptible (BC/S) families at below-label mesotrione dose (26 g ai ha^−1^).

**Run**	**Dose (g ai ha^−1^)**	**Family^a^**	**Plants treated**	**Survivors (observed)**	**Survival ratio**	**Survivors (expected)**	**χ^2^**	***P*-value**
1	26	R	10	10	1.00			
		S	8	6	0.75			
		SR/F1-8	13	13	1.00			
		SR/F1-9	9	9	1.00			
		SR/F1-13	8	8	1.00			
		F2 segregation				1R:2F1:1S		
		F2-9	75	65	0.87	70.3	6.4	0.01
		F2-13	98	98	1.00	91.8	6.6	0.01
		BC/S segregation				1F1:1S		
		BC-8	93	93	1.00	81.3	13.4	0.00
		BC-9	94	94	1.00	82.2	13.4	0.00
		BC-13	60	57	0.95	52.5	3.1	0.07
2	26	R	16	16	1.00			
		S	14	11	0.78			
		SR/F1-8	24	24	1.00			
		SR/F1-9	14	14	1.00			
		SR/F1-13	24	24	1.00			
		F2 segregation				1R:2F1:1S		
		F2-9	97	97	1.00	91.8	5.5	0.02
		F2-13	98	98	1.00	92.7	1.8	0.02
		BC/S segregation				1F1:1S		
		BC-8/S	29	27	0.97	25.9	0.8	0.38
		BC-9/S	95	95	1.00	84.8	11.4	0.00
		BC-13/S	49	48	0.98	43.7	5.9	0.04

*Chi-square (χ^2^) analysis for expected plant survival assuming control for mesotrione resistance by a single major gene*.

a *S, 4-hydroxyphenylpyruvate dioxygenase (HPPD)-inhibitor herbicide-susceptible A. tuberculatus collected from a field in Dixon County, NE in 2014; HPPD-R, 4-hydroxyphenylpyruvate dioxygenase (HPPD)-inhibiting herbicide-resistant A. tuberculatus collected from a field in Platte County, NE in 2014. SR/F1-8 (generated BC-8/S), SR/F1-9 (generated F2-9 and BC-9/S), and SR/F1-13 (generated F2-13 and BC-13/S) are crosses originated from SxR parents made under greenhouses conditions*.

**Figure 3:**
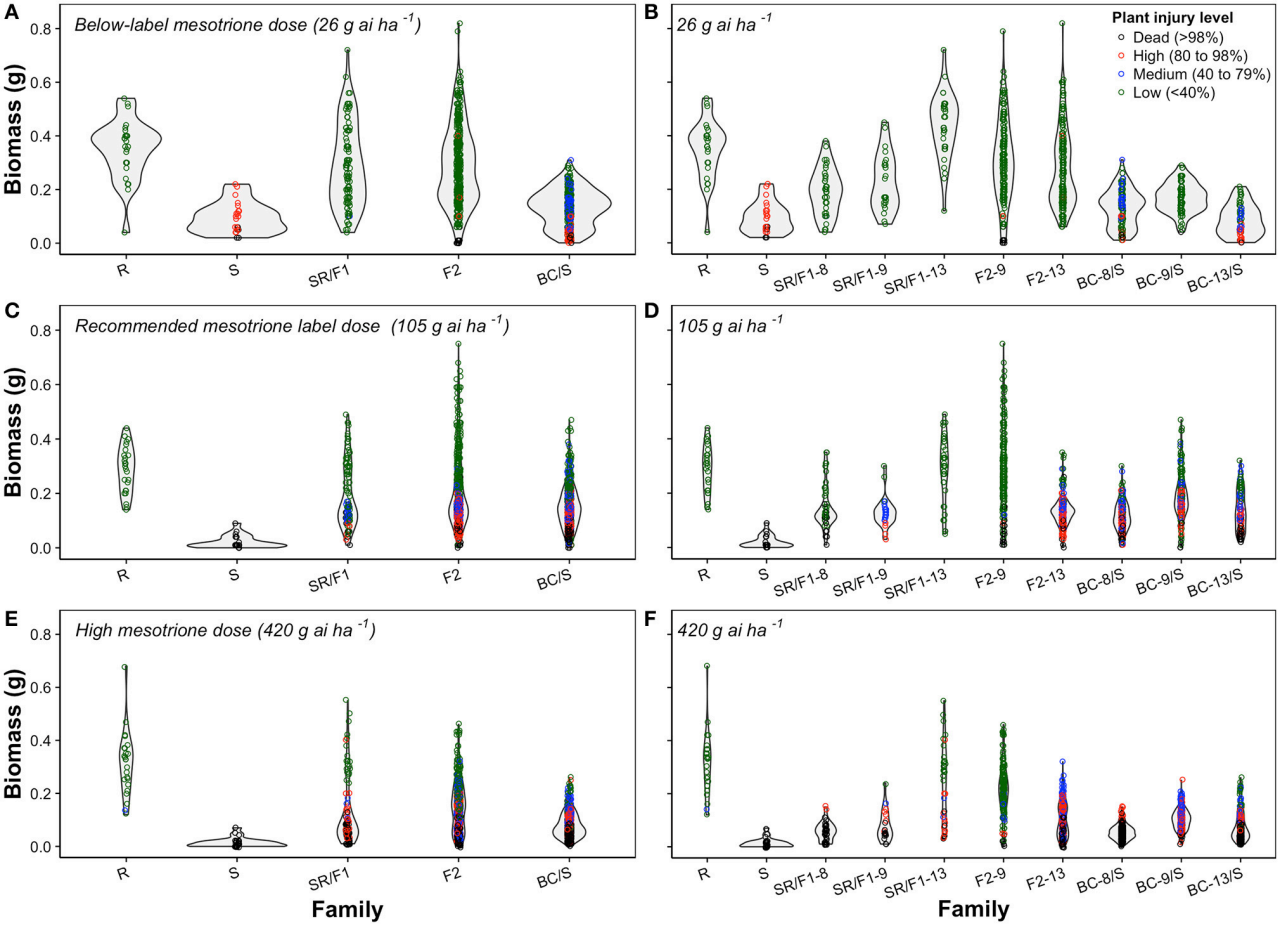
Violin plots combining a rotated kernel density plot on each side of mesotrione inheritance of *Amaranthus tuberculatus* families at below-label mesotrione dose (26 g ai ha^−1^) of combined **(A)** or separated families **(B)**; recommended mesotrione label dose (105 g ai ha^−1^) of combined **(C)** or separated families **(D)**; and high mesotrione dose (420 g ai ha^−1^) of combined **(E)** or separated families **(F)**. The dots represent biomass (g) for an individual plant within a population and colors denote the mesotrione injury level in experiment conducted at the University of Nebraska-Lincoln.

The recommended (105 g ai ha^−1^; Table 5) and a high mesotrione rate (420 g ai ha^−1^; Table 6) effectively distinguished R and S plant survival. At these two rates, R plants survived with low injury (< 40%), and S plants died (Figures 3C,E). However, F1 family survival and level of injury at 105 and 420 g ai ha^−1^ varied within families (Figures 3D,F). At mesotrione rate of 105 g ai ha^−1^, segregation in each family deviated from the corrected 1R:2F1:S (F_2_) and 1F1:1S (BC) ratios expected for one major gene in the two experimental runs (Table 5). Two and three loci segregation model tested fitted for most of F2 and BC/S, families (data not shown). For 420 g ai ha^−1^, only F2-13 (first run) and BC-8/S (second run) families did not deviate from the expected one major locus (Table 6). However, BC-8/S (first and second run), F2-13 (second run), and BC-13/S (first run) also did not deviate from two and three loci segregation (data not shown). In general, at 105 and 420 g ai ha^−1^ doses, the plant mortality was variable but higher than expected.

**Table 5:** Phenotypic resistance segregation observed in two pseudo-F2 and three back-cross susceptible (BC/S) families at recommended label mesotrione dose (105 g ai ha^−1^).

**Run**	**Dose (g ai ha^−1^)**	**Family^a^**	**Plants treated**	**Survivors (observed)**	**Survival ratio**	**Survivors (expected)**	**χ^2^**	***P*-value**
1	105	R	8	8	1			
		S	8	0	0			
		SR/F1-8	24	19	0.79			
		SR/F1-9	14	14	1			
		SR/F1-13	13	13	1			
		F2 segregation				1R:2F1:1S		
		F2-9	96	80	0.83	72.0	10.6	0.00
		F2-13	98	87	0.89	73.5	9.9	0.00
		BC/S segregation				1F1:1S		
		BC-8/S	96	85	0.89	37.9	96.7	0.00
		BC-9/S	96	90	0.94	48.0	73.5	0.00
		BC-13/S	49	40	0.82	24.5	19.6	0.00
2	105	R	17	17	1			
		S	13	0	0			
		SR/F1-8	24	13	0.55			
		SR/F1-9	10	10	1			
		SR/F1-13	24	23	0.96			
		F2 segregation				1R:2F1:1S		
		F2-9	88	85	0.97	66.0	21.9	0.00
		F2-13	95	91	0.96	69.3	25.1	0.00
		BC/S segregation				1F1:1S		
		BC-8/S	66	59	0.89	17.8	130.6	0.00
		BC-9/S	95	94	0.99	47.5	91.0	0.00
		BC-13/S	49	33	0.67	23.5	7.4	0.00

*Chi-square (χ^2^) analysis for expected plant survival assuming control for mesotrione resistance by a single major gene*.

a *S, 4-hydroxyphenylpyruvate dioxygenase (HPPD)-inhibitor herbicide-susceptible A. tuberculatus collected from a field in Dixon County, NE in 2014; HPPD-R, 4-hydroxyphenylpyruvate dioxygenase (HPPD)-inhibiting herbicide-resistant A. tuberculatus collected from a field in Platte County, NE in 2014. SR/F1-8 (generated BC-8/S), SR/F1-9 (generated F2-9 and BC-9/S), and SR/F1-13 (generated F2-13 and BC-13/S) are crosses originated from SxR parents made under greenhouses conditions*.

**Table 6:** Phenotypic resistance segregation observed in two pseudo-F_2_ and three back-cross susceptible (BC/S) families at high mesotrione dose (420 g ai ha^−1^).

**Run**	**Dose (g ai ha^−1^)**	**Family^a^**	**Plants treated**	**Survivors (observed)**	**Survival ratio**	**Survivors (expected)**	**χ^2^**	***P*-value**
1	420	R	8	8	1.00			
		S	8	0	0.00			
		SR/F1-8	13	1	0.08			
		SR/F1-9	9	3	0.33			
		SR/F1-13	12	9	0.75			
		F2 segregation				1R:2F1:1S		
		F2-9	91	86	0.95	37.9	104.6	0.00
		F2-13	96	56	0.58	60.0	0.7	0.40
		BC/S segregation				1F1:1S		
		BC-8/S	98	10	0.10	3.8	11.1	0.00
		BC-9/S	96	84	0.88	16.0	346.8	0.00
		BC-13/S	86	54	0.63	32.3	23.3	0.00
2	420	R	16	16	1.00			
		S	15	0	0.00			
		SR/F1-8	24	1	0.04			
		SR/F1-9	9	4	0.44			
		SR/F1-13	24	22	0.92			
		F2 segregation				1R:2F1:1S		
		F2-9	56	56	1.00	26.4	62.8	0.00
		F2-13	94	82	0.87	66.6	12.2	0.00
		BC/S segregation				1F1:1S		
		BC-8/S	91	0	0.00	1.9	1.9	0.16
		BC-9/S	94	68	0.72	20.9	136.5	0.00
		BC-13/S	85	20	0.24	38.9	16.9	0.00

*Chi-square (χ^2^) analysis for expected plant survival assuming control for mesotrione resistance by a single major gene*.

a *S, 4-hydroxyphenylpyruvate dioxygenase (HPPD)-inhibitor herbicide-susceptible A. tuberculatus collected from a field in Dixon County, NE in 2014; HPPD-R, 4-hydroxyphenylpyruvate dioxygenase (HPPD)-inhibiting herbicide-resistant A. tuberculatus collected from a field in Platte County, NE in 2014. SR/F1-8 (generated BC-8/S), SR/F1-9 (generated F2-9 and BC-9/S), and SR/F1-13 (generated F2-13 and BC-13/S) are crosses originated from SxR parents made under greenhouses conditions*.

The plant biomass (g) and injury levels (%) of the segregation analysis at different mesotrione doses on *A. tuberculatus* families was illustrated with combined (Figures 3A,C,E) or separated (Figures 3B,D,F) violin plots. The plant biomass of the generated families overlapped the R and S parent phenotypic ranges. The violin plots showed either continuous or bell-shaped distribution of biomass (g) across families and mesotrione rates (Figures 3A–F). For example, at 105 g ai ha^−1^ of mesotrione, the F2-9 family showed a majority of plants with low injury and highly variable biomass (Figure 3D). The F2-13 family had a medium to high injury with relatively low uniform biomass. Nonetheless, the F2 families resulted in high plant survival (Table 5). In contrast, the BC/S families resulted in a uniform relatively low biomass across families and mesotrione doses (Figures 3B,D,F). In addition, the shape of the phenotype was more continuous and thinner as the mesotrione dose increased (Figures 3A–F).

## Discussion

The individual plants representing the R population have been under continual selection pressure for mesotrione resistance, but they have not been through inbreeding that would increase homozygosity. The F1 families showed a lack of dominance or recessivity (Table 3), and the degree of dominance in F1 families varied from additive (SR/F1-5), incomplete recessive (SR/F1-9), and incomplete dominance (SR/F1-13; Table 3). The closest to complete dominance was SR/F1-13, which was the only F1 family for which the R parent was previously crossed under greenhouse conditions (R × R). Therefore, further inbreeding would be needed to establish families that could be used in future studies to test for allelism by crossing with other resistant populations and to further characterize the genotype × herbicide treatment environment.

The majority of weed species show either semi-dominance or dominance of inherited herbicide resistant alleles (Mallory-Smith et al., 1990; Lorraine-Colwill et al., 2001; Busi and Powles, 2017). For example, lack of dominance and high degree of genetic complexity within population has been documented in cross-pollinated (dioecious) weed species, including *Lolium rigidum, A. tuberculatus*, and *Alopecurus myosuroides* (Petit et al., 2010; Busi et al., 2013; Huffman et al., 2015). In contrast, the majority of insecticide resistance is recessive (Sandrock and Vorburger, 2011; Shen et al., 2017; Amusa et al., 2018). In theory, insect mating (S × R) would result in heterozygote susceptible (F1 families) phenotypes. Recessive inheritance was a key factor for the success of refugee strategy in Bt crops for delaying insecticide resistance evolution (Tabashnik et al., 2013; Jin et al., 2014). Therefore, tactics for combating herbicide resistance may be more complex than for insecticide resistance evolution. In our study, semi-dominance of mesotrione resistance was evident (Figures 2A,B). In such cases, theoretically, the heterozygote progeny (F1) would survive the field recommended herbicide dose (105 g ai ha^−1^).

Similar results from the reciprocal crosses (S × R and R × S) in F1 families indicated nuclear inherited resistance alleles (Figures 2A,B). As a result, nuclear inheritance allows seed and pollen movement carrying herbicide resistance alleles (Busi et al., 2011). Pollen-mediated gene flow plays an important role in dispersing herbicide resistance traits, which was previously documented by intra- and inter-specific hybridization studies in *Amaranthus* species (Gaines et al., 2012; Liu et al., 2012; Sarangi et al., 2017). This result highlighted the potential gene flow for spreading the metabolism-based mesotrione resistance from this *A. tuberculatus* population from Nebraska, and the likelihood to increase the frequency of resistance genes within the population (Jasieniuk et al., 1996).

Herbicide resistance traits in weeds are usually nuclear inherited (Jasieniuk et al., 1996; Gaines et al., 2012). In addition, our results support the conclusion that a single major gene did not control metabolism-based resistance in this *A. tuberculatus* population from Nebraska (Tables 4–6). Regardless of the apparent heterogeneity, there was no mortality in the R population at the three mesotrione doses applied. In fact, the R population is highly resistant as R plants survived 420 g ai ha^−1^ of mesotrione with low injury (Figure 3E). Even with the apparent lack of homogeneity in the R parent population, the variability of the phenotype in each generated family would not be explained by a single major gene (e.g., segregation pattern of BC and F2 families; Figures 3A–F). Also, mesotrione segregation analysis in the parent (R and S) populations and in the F1, F2, and BC/S families showed either continuous or bell-shaped distributions for *A. tuberculatus* biomass (Figures 3A–F), which is a typical response of quantitative (polygenic) traits (Morton and MacLean, 1974; Huffman et al., 2015). Furthermore, in our study, plant mortality was lower than expected at lower doses and higher than expected at higher doses (Tables 4–6), suggesting that inheritance of mesotrione resistance alleles is additive (Tabashnik, 1991).

In weeds, herbicide metabolism-based resistance is not well-understood and usually conferred by multiple alleles (Délye et al., 2013). For example, polygenic resistance was reported in NTSR mechanisms of ACCase- and ALS-inhibitor herbicides in *L. rigidum* populations (Busi et al., 2011, 2013; Han et al., 2014) and to ACCase-inhibitors in *Avena fatua* (Burns et al., 2018). However, monogenic resistance was found to endow NTSR mechanisms as well. A single major gene explained auxinic, glyphosate, and pyroxasulfone resistance in *L. rigidum* populations (Lorraine-Colwill et al., 2001; Busi et al., 2014; Busi and Powles, 2017) and atrazine resistance in *A. tuberculatus* (Huffman et al., 2015). Therefore, a single gene or multiple genes can endow NTSR mechanisms in weeds, suggesting that the number of alleles controlling NTSR varies according to the ecology and evolutionary factors contributing for weed resistance evolution. Also, these results demonstrated the complexity of NTSR mechanisms in weed species, which might be influenced by epigenetic effects (Yu and Powles, 2014; Markus et al., 2018).

Previous research in this R population suggested that HPPD-resistance is likely due to multiple cytochrome P450 enzymes, which is also indicative of polygenic resistance (Oliveira et al., 2017b). The cytochrome P450s comprise a large plant gene family and have repeatedly been associated with metabolism-based resistance in grass weed species (Powles and Yu, 2010). Examples of metabolism-based resistance via cytochrome P450s in grass weed species include *L. rigidum* (Christopher et al., 1991; Busi et al., 2011, 2013, 2014; Han et al., 2014); *A. myosuroides* (Letouzé and Gasquez, 2003); and *Echinochloa phyllopogon* (Yun et al., 2005; Iwakami et al., 2014). In dicotyledon species, metabolism-based resistance is still under-studied (Powles and Yu, 2010). In such cases, often the presence of TSR mechanisms might mask the metabolism-based resistance (Yu and Powles, 2014). Nonetheless, metabolism-based resistance via cytochrome P450s gained attention with the evolution of 2,4 D (Figueiredo et al., 2017) and HPPD resistance (Ma et al., 2013; Kaundun et al., 2017; Nakka et al., 2017) in *Amaranthus* species. Multiple genes were found to confer resistance in a metabolism-based mesotrione resistant *A. tuberculatus* population from Illinois (Huffman et al., 2015). Despite different genetic background, farming system, and potentially different cytochrome P450 enzymes causing resistance, the genetics of mesotrione resistance is consistent with polygenic inheritance in the Nebraska (Oliveira et al., 2017a) and Illinois (Huffman et al., 2015) *A. tuberculatus* populations. Therefore, there are likely multiple genes involved in mesotrione resistant-*A. tuberculatus* populations across the north-central United States.

In summary, we confirmed that mesotrione resistance in an *A. tuberculatus* from Nebraska is nuclear inherited and likely mediated by multiple genes. It remains unknown if another mechanism of herbicide resistance has arisen in the R population from Nebraska. Other proteins play an important role in herbicide compartmentalization (transporter proteins), degradation (glutathione-*S*-transferases, glycosyl-transferases, esterases, hydrolases), and compensation (oxidases, peroxidases; Délye, 2013; Ghanizadeh and Harrington, 2017). For example, other steps in herbicide metabolism in addition to oxidation by P450s, such as initial oxidation followed by conjugation (e.g., to a sugar) may be part of a metabolic resistance mechanism. The multiple metabolic steps would be consistent with additive inheritance, due to the requirement of upregulation of more than one gene to confer high level resistance and partial resistance conferred from upregulation of only one.

## Author contributions

MO and TG: designed the experiments; MO: conducted the experiments, analyzed the data, and wrote the manuscript; TG, SK, and AJ: conceptualized the research. All authors reviewed the manuscript.

### Conflict of interest statement

The authors declare that the research was conducted in the absence of any commercial or financial relationships that could be construed as a potential conflict of interest.
