# Supplementary material for: Inheritance of Mesotrione Resistance in an Amaranthus tuberculatus (var. rudis) Population from Nebraska, USA
Source: Front Plant Sci. 2018 Feb 2;9:60. doi: 10.3389/fpls.2018.00060 (PMC5801304; doi:10.3389/fpls.2018.00060)
Supplement: Supplementary file 1 [file SupplementaryMaterial.docx]

**Supplemental File**


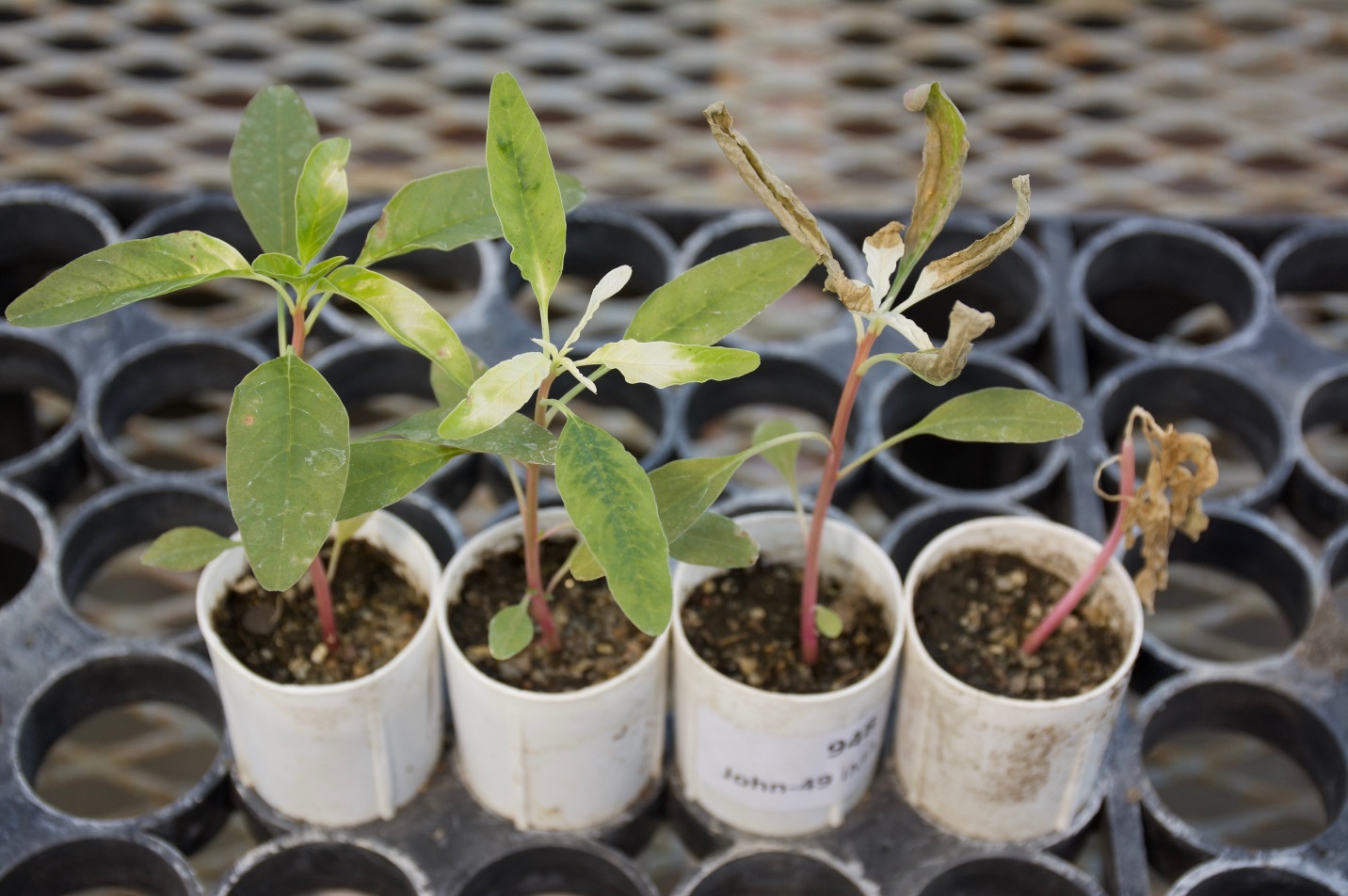


**Figure S1** *Amaranthus tuberculatus* phenotype of R and S parent populations; and F1 families, RS/F1-5, SR/F1-9, and SR/F1-13 evaluated 21 days after mesotrione application in the segregation analysis. From the left to right, low injury (<40%), medium injury (41 to 79%), high injury (80 to 98%), and dead plants (>98%)

**Table S1** Generations of F1, pseudo-F2 (F2), and backcross susceptible (BC/S) families in the *Amaranthus tuberculatus* population from Nebraska. The R and S population seeds were collected from multiple plants in Platte and Dixon Counties, NE, respectively. The R and S seeds were baged separately and used form the inheritance study. Reciprocal crosses was performed (R × S and S × R), and the R parent from family 13 was previously outcrossed (R × R).

| Parent | R (female) × S (male) | | S (female) × R (male) | |
| --- | --- | --- | --- | --- |
| F1 | RS/F1-5 |  | SR/F1-8 | SR/F1-9 |
| F2 |  |  |  | F2-9 |
| BC/S |  |  | BC-8/S | BC-9/S |
| Family # | 5 |  | 8 | 9 |
|  |  |  |  |  |
| Preliminary cross |  | R × R |  |  |
| Parent | S (female) × R (male) | |  |  |
| F1 | SR/F1-13 | |  |  |
| F2 | F2-13 | |  |  |
| BC/S | BC-13/S | |  |  |
| Family # | 13 | |  |  |

**Table S2** Genotypic segragation pattern and expected phenotypic survival in F2 and BC/S families to one locus (A), two loci (B), and three loci segregation (C) resistance models. The resistant and susceptible alleles are represented by *R* and *r*, respectively. The absence of the *R* (resistant allele) does not endow mesotrione resistant in *Amaranthus tuberculatus* and the genotypic combinations are highlighted in red color. The genotype combinations highlighted in yellow color indicate the presence of resistant allele (*R1*, *R2*, and *R3*). The genotypes combinations in green color specify resistance endowed similar in the parental resistant line.

| A) One locus segregation model for F_2_ and BC. | | |
| --- | --- | --- |
| **Alleles** | *R1* | *r1* |
| *R1* | *R1R1* | *R1r1* |
| *r1* | *r1R1* | *r1r1* |
| F2 Genotypic Segregation 1R:2F_1_:1S | | |
|  |  |  |
|  |  |  |
| Alleles |  | *r1* |
| *R1* |  | *R1r1* |
| *r1* |  | *r1r1* |
| BC/S Genotypic Segregation: 1F1:1S | | |

Example referred to Table 4 in the manuscript for one major locus segregation:

**Mesotrione dose at 105 g ai ha^-1^**

- *F2 population (first run) – Family F2-9*

Exp F2 = 0.25 × Obs R + 0.5 × Obs F1 + 0.25 × Obs S

Exp F2 = 0.25 × 1 + 0.5 × 1 + 0.25 × 0

Exp F2 = 0.75 × 96 (total plants of F2-9 family)

Exp F2 = 72 plants

- *BC population (first run) – Family BC-9*

Exp BC = 0.5 × (Obs F1 + Obs S)

Exp BC = 0.5 × (1 + 0)

Exp BC = 0.5 × 96 (total plants of BC-9 family)

Exp BC = 48
